# Editorial: Apatinib and Anlotinib in the Treatment of Radioactive Iodine Refractory and Highly Invasive Thyroid Carcinoma

**DOI:** 10.3390/jcm11216380

**Published:** 2022-10-28

**Authors:** Wenqing Jia, Zhuoran Liu, Ling Zhan, Qiwu Zhao, Weihua Qiu, Jie Kuang

**Affiliations:** Department of General Surgery, Ruijin Hospital, Shanghai Jiao Tong University School of Medicine, Shanghai 200025, China

Thyroid cancer (TC) is the most prevalent endocrine malignancy, with a rising incidence in the past decade. Based on the derivation of neoplasms, TC comprises thyroid follicular epithelial-derived cancers and parafollicular cell (also called C cells)-derived cancer (MTC), while the former can be further divided into differentiated thyroid cancer (DTC), poorly differentiated thyroid carcinoma (PDTC), and anaplastic thyroid carcinoma (ATC).

Although DTC is a common subtype, representing about 90% of TC, with a comparatively ideal prognosis in response to standard therapies, including surgical resection and chemical as well as isotopic interventions, it has been validated as bona fide that some patients progress to radioactive iodine refractory differentiated thyroid carcinoma (RAIR-DTC) [1]. MTC, PDTC, and ATC account for less than 10% of TC, but are fatal malignancies leading to nearly a quarter of thyroid-cancer-related deaths, partially owing to characteristics that include rapid proliferation, early dissemination, and distant metastasis [2,3,4]. Canonical therapies, such as surgery and ^131^I intervention, fail to demonstrate therapeutic effects on RAIR-DTC and other highly invasive thyroid carcinoma. Therefore, state-of-art therapies such as targeted drugs and immunotherapeutic agents must urgently be fully enumerated and further explored.

**Apatinib in the treatment of RAIR-DTC and highly invasive thyroid carcinoma.**

Tumor-associated angiogenesis is among the 14 hallmarks of cancer [5,6], in which the vascular endothelial growth factor (VEGF) pathway plays a leading role. The overexpression of the VEGF receptor (VEGFR), including VEGF-A, VEGFR1, and VEGFR2, can be observed in more than 90% of TC patients and correlates with poor prognosis. The activation of VEGFR promotes the proliferation, survival, and migration of epithelial cells, thereby increasing vascular permeability, and promotes tumor angiogenesis by activating the PI3K/Akt pathway [7,8]. In recent years, significant progress in antiangiogenic agents has been witnessed in the treatment of RAIR-DTC and highly invasive thyroid cancer [9,10]. However, further endeavors are expected to be carried out to overcome complexities such as unaffordable prices, severe adverse effects, unstable efficacies, and unknown drug resistance mechanisms.

Apatinib can selectively block VEGFR2 [11], suppressing the VEGF-stimulated migration and proliferation of vascular endothelial cells, reducing tumor microvessel density, and inhibiting tumor progression as well as metastasis [12,13]. Studies have confirmed the efficacy and safety of apatinib in treating malignancies with rich blood supply [14]. Additionally, the application of apatinib has been expanded to advanced gastric cancer, non-small-cell lung cancer (NSCLC), breast cancer, and hepatocellular carcinoma, according to several phase II and III clinical trials [15,16,17,18,19]. As a therapy for RAIR-DTC and highly invasive thyroid cancer, apatinib has displayed preliminary efficacy in several clinical contexts.

One of the important pathological features of RAIR-DTC and highly invasive thyroid cancer is rapid and severe local invasion to nearby organs, such as the recurrent laryngeal nerve, trachea, esophagus, larynx, pharynx, or major vessels in the neck. The primary lesion rapidly evolves into an unresectable tumor, making radical surgery and even palliative tumor reduction surgery therapeutic challenges. It has been proven that apatinib can be used as a neoadjuvant agent since it is effective in reducing tumor load and relieving local compression symptoms, thus raising the number of resectable cases [20]. 

Lin et al. [21] conducted a trial involving 10 RAIR-DTC patients who were treated with apatinib. The disease control rate (DCR) was 100%, and the objective remission rate (ORR) was 90%, which is higher compared with the previously reported results of sorafenib or lovatinib [9,10,21]. According to a recent network meta-analysis on multiple targeted therapeutics available for RAIR-DTC, apatinib was associated with the best overall survival (OS) benefits [22].

The efficacy of radiotherapy and chemotherapy for advanced MTC is limited, and the use of the VEGFR inhibitor apatinib may become an alternative choice for the treatment of advanced MTC. Cai et al. [2] reported a case of apatinib successfully treating the postoperative recurrence of MTC with hilar, mediastinal lymph nodes and bilateral lung metastasis, with significantly declining serum carcinoembryonic antigen (CEA) and calcitonin concentrations as well as the shrinkage of pulmonary metastases. Another MTC patient with extensive metastasis, who failed to respond to a 1.5-year period of targeted therapy with sorafenib (400–600 mg/d), achieved partial remission after apatinib treatment for 16 weeks and lasted for more than 9 weeks [23]. 

All ATC cases are classified as stage IV TC by the American Joint Cancer Commission [24]. Patients with stage Ⅳb and Ⅳc ATC find it difficult to benefit from radical resection, including laryngectomy and esophagectomy [25,26]. Niu et al. [27] and Cheng et al. [3] depicted the possibility of apatinib in treating ATC patients. 

Despite the conspicuous and promising role of apatinib bursting onto the scene of RAIR-DTC and highly invasive thyroid cancer, several deficiencies are expected to be refined. Since the underlying molecular mechanism and related signal pathway have been gradually elaborated on, multiple combined therapies are under exploration with the intention of maximizing sensitivity and minimizing toxicity.

Apoptosis and autophagy play significant roles in regulating cell survival and balancing the homeostasis of the intracellular microenvironment. Our team revealed that apatinib reduces the expression of angiopoietin by inhibiting the Akt/GSK3β/ANG signal pathway, thus inhibiting angiogenesis in the tumor microenvironment of ATC. It also causes a halt in the cell cycle at the G0/G1 phase, thus inducing tumor cell apoptosis, and restricting its proliferation in a dose- and time-dependent manner [28]. Apatinib can also downregulate the p-Akt and p-mTOR signals via the Akt-mTOR pathway, further inducing the autophagy and apoptosis of ATC cells. Moreover, the inhibition of apatinib-induced autophagy increased apatinib-induced apoptosis in ATC cells. Therefore, the combination of apatinib and autophagy inhibitors shows promise as an improvement in treatment [29,30].

The combination of apatinib and other therapies, such as chemotherapy, radiotherapy, or other targeted agents, demonstrates a synergistic antitumor effect, which has been confirmed through in vivo and in vitro studies [31]. In the mouse model of nasopharyngeal carcinoma, it was observed that the expression of VEGFR2 was significantly reduced, the apoptosis rate was increased, and the inhibition of tumor growth was enhanced, leading to prolonged survival time after treatment with apatinib and cisplatin [32]. The antitumor effect of cisplatin on the thyroid papillary carcinoma cell (TPC-1) was further enhanced through the VEGFR2-Akt-mTOR pathway after treatment with apatinib [29]. Li et al. [33] found that apatinib can significantly enhance the antitumor effect of gefitinib on NSCLC resistant to epidermal growth factor receptor (EGFR) tyrosine kinase inhibitors by activating the EGFR as well as VEGFR2 and downregulating the expression of CD31 and VEGF-A. 

Multidrug resistance (MDR) refers to broad resistance to a variety of antitumor agents with different structures and mechanisms, which is a major obstacle in consistently beneficial treatments of tumors. The ABC transporter family is considered a group of MDR transporters in tumor cells of great significance, the overexpression of which is closely associated with worse chemotherapy responses and lower OS of cancer populations. Antagonists of the ABC transporter can re-sensitize MDR cancer cells’ chemotherapy. Studies have shown that apatinib can reverse ABCB1-mediated MDR in tumor cells through the direct blockage of ABCB1, increasing the intracellular concentration of chemotherapy drugs, thus enhancing the antitumor effect of chemotherapy agents. Therefore, the combined application of apatinib and anticancer drugs is very promising in overcoming chemoresistance [34,35].

**Anlotinib in the treatment of RAIR-DTC and highly invasive thyroid carcinoma.**

Anlotinib is a multi-targeted tyrosine kinase inhibitor that inhibits both tumor angiogenesis and tumor cell proliferation by blocking the vascular endothelial growth factor receptors (VEGFR1, VEGFR2/KDR, and VEGFR3), fibroblast growth factor receptors (FGFR1, FGFR2, and FGFR3), platelet-derived growth factor receptors (PDGFR), and c-Kit simultaneously [36]. It has been approved by the China National Medical Products Administration (NMPA) as a novel third-line therapeutic option for advanced non-small-cell lung cancer (NSCLC) in 2018 [37], and is also effective in treating metastatic renal cell carcinoma as well as bone and soft tissue sarcoma [38]. Its recommended schedule is 12 mg p.o. daily with a 2-week on/1-week off regimen [39].

The sustained antitumor activity of anlotinib was proven in locally advanced or metastatic MTC, with a PFS rate of 85.5% at 48 weeks of treatment according to a phase II trial conducted by Sun et al. [40]. Li et al. [41] also demonstrated the efficacy and safety of anlotinib in ALTER 01031, a multicenter phase IIB clinical trial (NCT02586350) which enrolled 91 Asian patients with histopathologically confirmed, unresectable locally advanced or metastatic MTC. The median PFS was significantly prolonged in the anlotinib group compared to the placebo group (20.7 months vs. 11.1 months, *p* = 0.029, 95% CI 0.30–0.95). 

As for radioactive iodine refractory differentiated thyroid cancer (RAIR-DTC), a phase II clinical trial (NCT02586337) involving 113 patients was conducted in China [42], the results of which revealed a significant prolonged PFS of the anlotinib group (40.5 months vs. 8.4 months, *p* < 0.001) and an ORR of 59.21%. One of the cases with recurrent and metastatic RAIR-DTC achieved over 37 months of PFS, and it was noteworthy that the patient demonstrated a coexistent C228T mutation of the TERT promoter and BRAF V600E mutations [43]. 

It is also possible that anlotinib can benefit populations with anaplastic thyroid carcinoma (ATC). Clinically, anlotinib, as an anti-angiogenesis agent, combined with the immunotherapeutic agent sintilimab, depicted astonishing effects in treating an ATC patient, leading to remarkable tumor shrinkage and an 18.3-month sustained remission period [44]. Considering that this patient carried none of the mutations indicated as corresponding to targeted therapy but had high PD-L1 expression (TPS 60%), the PD-1 inhibitor plus anlotinib as a first-line therapy deserve further investigation in ATC populations. Mechanically, our team confirmed that the EGFR is a novel target of anlotinib, and phosphor-EGFR, rather than other known mechanisms, was downregulated in ATC cells treated with anlotinib [45]. Furthermore, we revealed that anlotinib blocked intercellular crosstalk via a dual mechanism through simultaneous inhibitory effects on cancer cells and endothelium.

Besides focusing on targeted therapy for unresectable thyroid cancer, researchers also explored the manifestation of anlotinib in the neoadjuvant setting, targeting locally advanced thyroid cancer. Huang et al. [46] reported in a single-arm phase II trial (NCT04309136) that the objective response rate (ORR) of anlotinib was 76.9% (95% CI 46.2–95.0%), while the R0/R1 resection rate in the intent-to-treat population was 61.5% and in the per-protocol population was 72.7%.

Apatinib and anlotinib are of acceptable and manageable safety. Most adverse reactions are mild to moderate [18]. The common adverse events include hand–foot syndrome and hypertension at dose-limiting toxicity [47]. Multiple dimensions of pharmacology, such as the ideal starting dose, adverse reaction monitoring, and treatment window as well as dose correction, and other treatment modes still deserve investigation in future.

In view of the poor prognosis and limited treatment options of RAIR-DTC and highly invasive thyroid cancer, the preliminary clinical study results of apatinib and anlotinib provide new alternatives. On this basis, further studies on the antiangiogenic mechanism of apatinib and promising targets of anlotinib are encouraged. A better therapy agenda is also expected to be established to minimize adverse reactions and drug resistance. We continue to foresee the development of apatinib and anlotinib in the treatment of thyroid cancer, and also anticipate that the signaling circuitry describing the intercommunication between various cells within tumors will be charted in far greater detail and clarity, eclipsing our current knowledge.
